# PRC2 regulates RNA polymerase III transcribed non-translated RNA gene transcription through EZH2 and SUZ12 interaction with TFIIIC complex

**DOI:** 10.1093/nar/gkv574

**Published:** 2015-06-01

**Authors:** Chang Liu, Shuai Li, Xiaoyan Dai, Ji Ma, Junhu Wan, Hao Jiang, Peng Wang, Zhaoli Liu, Hongquan Zhang

**Affiliations:** 1 Key Laboratory of Carcinogenesis and Translational Research, Ministry of Education and State Key Laboratory of Natural and Biomimetic Drugs, Peking University Health Science Center, #38 Xue Yuan Road, Beijing 100191, China; 2 Laboratory of Molecular Cell Biology and Tumor Biology, Department of Anatomy, Histology and Embryology, School of Basic Medical Sciences, Peking University Health Science Center, #38 Xue Yuan Road, Beijing 100191, China

## Abstract

Polycomb repression complex 2 (PRC2) component EZH2 tri-methylates H3K27 and exerts epigenetic repression on target gene expression. EZH2-mediated epigenetic control of RNA polymerase II (Pol II) transcribed coding gene transcription has been well established. However, little is known about EZH2-mediated epigenetic regulation of RNA polymerase III (Pol III) transcription. Here we present a paradigm that EZH2 is involved in the repression of Pol III transcription via interaction with transcriptional factor complex IIIC (TFIIIC). EZH2 and H3K27me3 co-occupy the promoter of tRNA^Tyr^, 5S rRNA and 7SL RNA genes. Depletion of EZH2 or inhibition of EZH2 methyltransferase activity led to upregulation of Pol III target gene transcription. EZH2-mediated repression of Pol III transcribed gene expression requires presence of SUZ12. SUZ12 was able to interact with TFIIIC complex and knockdown of SUZ12 decreased occupancy of EZH2 and H3K27me3 at the promoter of Pol III target genes. Our findings pointed out a previously unidentified role of PRC2 complex in suppressing transcription of Pol III transcribed non-translated RNA genes, putting Pol III on a new layer of epigenetic regulation.

## INTRODUCTION

Polycomb group proteins are important epigenetic regulators that play crucial roles in embryonic development and differentiation (1,2). EZH2 (Enhancer of Zeste Homolog 2) is a member of polycomb protein family, which forms PRC2 complex (Polycomb repression complex 2) with other polycomb members including SUZ12 and EED (3). Recent studies have identified an additional component of PRC2 in ESC cells, Jarid2, which recruits PRC2 to chromatin together with non-coding RNAs (4,5). EZH2 is the core catalytic component of PRC2, mediating trimethylation of histone H3 at lysine 27 (H3K27) through the SET domain, thus repressing the transcription of target genes (6,7). EZH2 was also found to participate in processes independent of PRC2 complex. It was found that phosphorylated EZH2 was able to activate STAT3 signaling through increased tyrosine phosphorylation of STAT3 via direct methylation of it, meanwhile, EZH2 could regulate gene transcription by integrating with estrogen and Wnt signaling as a coactivator (8,9). In addition, the oncogenic role of EZH2 in castration-resistant prostate cancer was found independent of PRC2 complex (10).

Overexpression of EZH2 is associated with progression of various tumors, including prostate and breast cancers (11,12). Recently, a number of EZH2 targets including E-cadherin, RUNX3, STAT3 and various EZH2 interacting proteins including Jarid2 and PHF1 have been found to mediate EZH2-regulated cancer progression (5,8,13–15). However, the function of EZH2 in cancer progression is still incompletely understood.

Pol III is responsible for the transcription of a series of small non-translated RNAs including transfer RNA (tRNA), the smallest subunit of ribosome (5S rRNA) and 7SL RNA. Transcriptional factors of Pol III contain TFIIIA, TFIIIB and TFIIIC. (16). A variety of proteins that were involved in regulation of Pol III transcription have been identified. C-Myc was found to interact with TFIIIB and robustly trigger Pol III transcription through recruitment of histone acetyltransferases TRRAP and GCN5 (17,18). It was also found that polo-like kinase PLK1 could regulate Pol III transcription through binding and phosphorylating Brf1, a subunit of TFIIIB (19). In contrast with the oncogenic protein c-Myc, tumor suppressive proteins including p53, PTEN, Rb and Maf1 were found to repress Pol III transcription through targeting or interacting with TFIIIB (20–24). In addition, mTOR was found present at the gene promoters of tRNA and 5S rRNA and affects their transcription through association with TFIIIC and their repressor Maf1 (25).

The fast development of deep sequencing technology allows large scale detection of modifications for genes coding small RNAs, including those transcribed by Pol III. ChIP-seq data mining in several studies suggests that modulation of Pol III Histone marks that were used to be found at promoters of Pol II transcribed genes are also present at promoters of Pol III target genes (26–28). Resembling Pol II transcribed genes, transcription of genes by Pol III exhibit a negative correlation with heterochromatic histone modifications and a positive correlation with euchromatic modifications. We present here that PRC2 members including EZH2 and SUZ12 are present at the promoters of the small non-translated RNA genes possibly through direct interaction with TFIIIC complex, a previously unidentified mechanism.

## MATERIALS AND METHODS

### Cell culture and treatment

The human cervical cancer cells HeLa and breast cancer cells MCF7, MDA-MB-231 and SUM159 were obtained from ATCC (American Type Culture Collection). All cells were grown in RPMI 1640 or Dulbecco's modified Eagle's medium supplemented with 10% fetal bovine serum (FBS) and cultured at 37°C with 5% CO_2_. SUM159 and MDA-MB-231 cells were treated with DZNep (Millipore) at different concentrations from 1 to 10 μM for 24 to 48 h before harvest.

### Plasmid construction and transfection

The pCMV6-Myc-DKK-GTF3C3 expression plasmid was purchased from OriGene (Rockville, MD, USA). The Flag-EZH2 plasmid was kindly provided by Professor Wei-guo Zhu (Peking University). The Flag-EZH2ΔSET plasmid was obtained by cloning the N-terminal 609 amino acids into the 3× Flag expression vector (Sigma). All constructs were confirmed by DNA sequencing. Transfections were performed using Lipofectamine 2000 (Invitrogen) according to the manufacturer's instruction. For transient gene silencing, small interfering RNAs were transfected into cells using RNAimax transfection reagent (Invitrogen) according to the standard protocol. Target sequences for transient silencing were: 5′-GCUGGGACAUGUACAAUGU-3′ (siRNA 1) and 5′-GGUCGCAGAUGUGUAUAAU-3′ (siRNA 2) for EZH2, 5′-GUCGCAACGGACCAGUUAA-3′ (siRNA 1) and 5′-GACUACAGAUCUACAAACA-3′ (siRNA 2) for SUZ12.

### Western blot and antibodies

Cells were lysed with NP-40 buffer (50 mM Tris–HCl, pH 7.4, 150 mM NaCl, 1% NP-40, 1 mM ethylenediaminetetraacetic acid (EDTA)). Antibodies against actin (Zhongshan Golden Bridge Biotechnology), EZH2 (Cell Signaling Technology #9441), TFIIIC102 (Santa Cruz sc-101176), TFIIIC110 (Santa Cruz sc-81406), TFIIIC63 (Santa Cruz sc-134082), SUZ12 (CST #3737), H3 (CST #9715), H3K27me3 (Abcam ab6002) were purchased from commercial sources.

### Identification of EZH2 interacting proteins by mass spectrometry

To identify new EZH2 interacting proteins, co-immunoprecipitation (Co-IP) assays were performed using EZH2 (CST, #9441) antibody and normal rabbit IgG. Proteins co-immunoprecipitated by EZH2 antibody were separated by sodium dodecyl sulphate-polyacrylamide gel electrophoresis (SDS-PAGE) and then stained by Coomassie blue. The corresponding bands were subjected to in-gel trypsin digestion. The labeled peptides were analyzed by LC-MS/MS using Nano-LC combined with Orbitrap Q Exactive mass spectrometer (Thermo Scientific, Grand Island, NY, USA).

### Co-immunoprecipitation

MCF7 and SUM159 cells were lysed with NP-40 buffer and lysates were incubated with relevant antibodies overnight at 4°C. Normal rabbit or mouse IgG was used as negative controls. Then 50 μl protein A/G-Sepharose was added to the mixture and incubated for 2 h. The immunoprecipitated complexes were washed with NP-40 buffer for 4× and then subjected to SDS-PAGE and immunoblot analysis with the indicated antibodies. The antibodies used for immuoprecipitation assays were EZH2 (CST #4905), SUZ12 (CST #3737), TFIIIC102 (Santa Cruz sc-101176) and TFIIIC110 (Santa Cruz Santa Cruz sc-81406).

### GST pull-down assays

The DNA fragments for the N-terminal region (1–522), Cys-Rich (523–609) domain and SET domain (610–746) of EZH2 were amplified with polymerase chain reaction (PCR). To obtain the glutathione S-transferase (GST) fusion proteins of EZH2, the above DNA fragments were subcloned into pGEX-4T-1 vector (GE Healthcare). The GST fusion proteins of TFIIIC102 were obtained with similar methods. GST and GST fusion proteins were expressed in *Escherichia coli* BL21 and purified with Glutathione Sepharose 4B beads (Pharmacia Biotech). Then the Glutathione Sepharose 4B beads and the purified proteins were mixed with lysates of HeLa cells transfected with Flag-TFIIIC102 or Flag-EZH2 plasmids and then incubated at 4°C over night. After being washed for 4× by NP-40 buffer, the co-immunoprecipitated proteins were subjected to western blot analysis.

### ChIP assays

MCF7, SUM159 or MDA-MB-231 cells (1 × 10^7^) were treated with 1% formaldehyde for 10 min at 37°C and then 0.125M glycine was added and incubated for 5 min at room temperature. Cells were lysed with lysis buffer (50 mM Tris–HCl, pH 7.4, 150 mM NaCl, 1% NP-40, 1 mM EDTA) on ice. The cell lysates were sonicated on ice for 10× with 6 s duration each time and the length of the DNA fragments were controlled at about 200–1000 bp. After centrifugation at 14 000 rpm for 15 min at 4°C, the lysates were diluted with nine volume of dilution buffer (1% Triton X-100, 2 mM EDTA, 50 mM NaCl, 20 mM Tris–HCl at pH7.9 and protease inhibitor cocktail). A total of 100 μl of the diluted lysate was spared as input and stored at 4°C until the step for reverse cross-linking. Immunoprecipitation was carried out with antibodies specific to EZH2 (Millipore), H3K27me3 (Abcam) and normal mouse IgG as a control and incubated overnight at 4°C. After immunoprecipitation, 20 μl magnet beads (Invitrogen) were added and incubated for 2 h. The magnet beads were then sequentially washed with TSE I (0.1% SDS, 1% Triton X-100, 2 mM EDTA, 150 mM NaCl, 20 mM Tris–HCl at pH 8.1), TSE II (0.1% SDS, 1% Triton X-100, 2 mM EDTA, 500 mM NaCl, 20 mM Tris–HCl at pH 8.1) and TSE III (0.25 M LiCl, 1%NP-40, 1% deoxycholate, 1 mM EDTA, 10 mM Tris–HCl at pH 8.1), and washed twice with TE buffer before being eluted with the elution buffer (1% SDS and 0.1 M NaHCO3). The eluates were incubated for 6–8 h at 65°C to reverse the formaldehyde cross-linking. DNA fragments were purified by using the PCR Purification Kit (Qiagen). Quantitative PCR (qPCR) was performed using Roche reagents. Each value was normalized to its respective input. The specific PCR primer pairs for ChIP were described in Supplementary Table S2.

### Quantitative PCR

Total RNA was extracted with TRIzol reagent (Invitrogen) according to the standard protocol and 2 μg total RNA was used for cDNA synthesis with random primers (Genestar). The cDNA was added to a qPCR mixture that contained 2× SYBR Green PCR master mixes (Roche) and 10 μM gene-specific primers. Assays were performed in triplicate. The PCR steps included incubations for 10 min 95°C, followed by 40 cycles, each cycle consisting of 15 s at 95°C and 1 min at 60°C. The expression levels of snRNAs were normalized with acidic ribosomal phosphoprotein P0 (ARPP P0). Primers used were described in Supplementary Tables.

### Analysis of histone marks on active and inactive tRNA genes by the ENCODE database

ChIP-seq dataset of histone marks including H3K4me2, H3K4me3, H3K9ac, H3K9me3 and H3K27me3 was acquired from Encyclopedia of DNA Elements (ENCODE) at UCSC (http://genome.ucsc.edu/). The ChIP signaling peaks between −1000 to +1000 from the TSS (Transcription start site) of the randomly chosen tRNA genes were captured from USCS genome browser and drawn into graphs.

### Statistical analysis

All data are presented as means ± standard deviations (SD) of results from three independent experiments. Statistical significance was determined by the two-tailed Student *t*-test. *P* < 0.05 was considered statistically significant.

## RESULTS

### EZH2 interacts with TFIIIC102 in breast cancer cells

**Figure 1.**
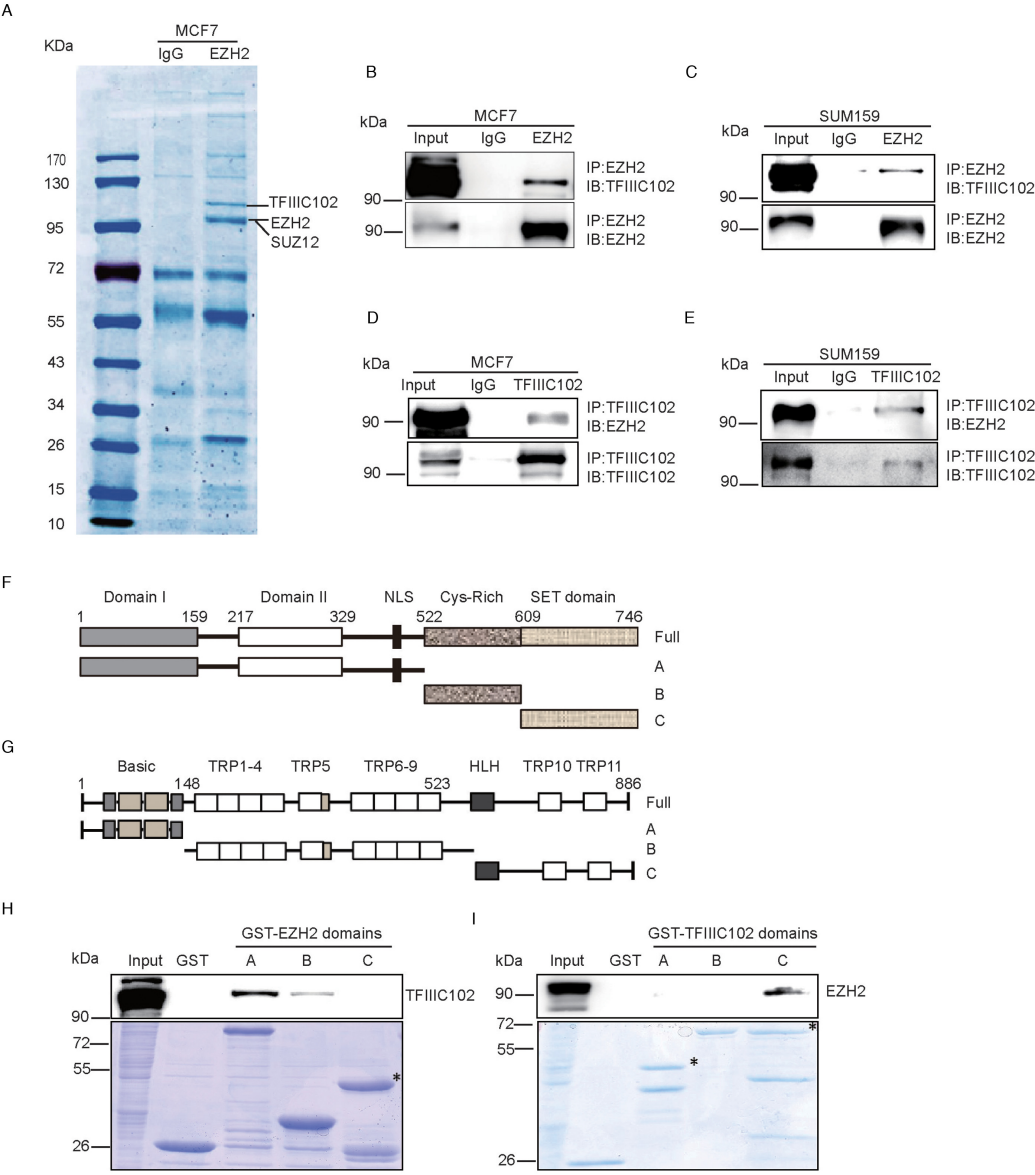
EZH2 interacts with TFIIIC102 in breast cancer cells. (**A**) SDS-PAGE gel showing proteins co-immunoprecipitated by IgG and an EZH2 antibody from MCF7 cells. The gel was stained by Coomassie brilliant blue and differential bands were subjected for mass spectrometry. Bands indicated showed TFIIIC, EZH2 and SUZ12 after determination by mass spectrometry. (**B** and **C**) Co-IP assays performed with an EZH2 antibody and analyzed by western blot using lysates from MCF7 and SUM159 cells with indicated antibodies. (**D** and **E**) Reverse Co-IP with TFIIIC102 antibody and analyzed by western blot using lysates from MCF7 and SUM159 cells with indicated antibodies. Rabbit or mouse IgG was used as negative controls. (**F**) Organization of the EZH2 functional domains. Three GST–EZH2 fragments were constructed: A. N-terminal region (Domain I + Domain II): aa 1–522. B. Middle region (Cys domain): aa 523–605. C. C-terminal region (SET Domain): aa 606–746. (**G**) Organization of the domains of TFIIIC102. Three GST–TFIIIC102 fragments were constructed: A.N-terminal region: aa 1–148. B. Middle region: aa 149–523. C. C-terminal region: aa 524–886. (**H**) GST pull-down assays were performed with purified GST–EZH2 fragments and HeLa cell lysates. Protein interaction was detected by western blot with TFIIIC102 antibody. The asterisk indicates the fragment C of GST–EZH2. (**I**) GST pull-down assays using GST–TFIIIC102 fragments and HeLa cell lysates were performed. Protein interaction was detected by Western blot with an EZH2 antibody. The asterisks indicate fragments A and C of GST–TFIIIC102 separately. GST alone was used as a negative control.

EZH2 is an important methyltransferase that is frequently overexpressed in breast and prostate cancers and is associated with tumor growth and invasion (11,12,29). Although a variety of EZH2 target genes and interacting proteins have been identified, new EZH2-mediated functions are still being explored (15,30). In an attempt to identify new EZH2 interacting proteins, co-immunoprecipitation (Co-IP) assays were performed with an EZH2 antibody in breast cancer cells MCF7. Protein co-immunoprecipitated with EZH2 was examined by Coomassie-stained gel and was further analyzed by mass spectrometry. Tryptic peptides identified by mass spectrometry revealed a panel of new EZH2 associated proteins (Figure 1a). Among the associated proteins, SUZ12 was co-immunoprecitipated with EZH2 antibody, indicating the correctness of the system (Figure 1a). Interestingly, TFIIIC102, a component of Pol III transcription factor C complex, displayed a high score in protein association and was selected for further characterization. To this end, we confirmed the interaction of endogenous EZH2 with TFIIIC102 by Co-IP with an EZH2 antibody from CST (Figure 1b) and re-examined it with an alternative antibody from Millipore (Supplementary Figure S1a). Accordingly, we identified the interaction between EZH2 and TFIIIC102 in another breast cancer cell line SUM159 (Figure 1c), suggesting that the interaction between EZH2 and TFIIIC102 occurs widely in breast cancer cells. In addition, we performed IP-western analysis with DNase which showed the interaction between EZH2 and TFIIIC102 was not DNA dependent (Supplementary Figure S1b). To re-examine the correctness of the protein interaction we performed a reverse Co-IP with TFIIIC102 antibody in MCF7 and SUM159 cells and demonstrated again that the interaction between the two proteins does exist (Figure 1d and e).

To map the binding regions between EZH2 and TFIIIC102, EZH2 was divided into three fragments and fused with GST separately (Figure 1f). Fragment A encodes the N-terminal domain I and domain II of EZH2, which has been found to interact with several proteins (9,31). Fragments B and C encode EZH2 Cys and SET domains separately. The three fragments were mixed individually with lysate of HeLa cells transfected with Flag-TFIIIC102 expression vector. GST pull-down assays demonstrated that EZH2 interacted with TFIIIC102 mainly through the N-terminal domain I and domain II. The interaction between the Cys domain of EZH2 and TFIIIC102 was weak, while the SET domain did not interact with TFIIIC102 (Figure 1h). These GST fusion proteins of EZH2 were previously used in GST pull-down assays in another study of our lab, in which fragment C was found to interact with PCAF (p300/CBP-associated factor), suggesting that all the three fragments were functional proteins (32). Similarly, TFIIIC102 was also divided into three fragments and only the C-terminal fragment C (aa 524–886) was capable of interacting with EZH2 (Figure 1g and i). To rule out the possibility that adapter proteins mediate the interaction between EZH2 and TFIIIC102, we performed *in vitro* pull down assays using purified proteins including fragment A (aa 1–522) of EZH2 fused with GST and fragment C (aa 524–886) of TFIIIC102 fused with MBP. It was demonstrated that GST-EZH2 (aa 1–522) interacted directly with the C-terminal fragment C of TFIIIC102 (Supplementary Figure S1c). Collectively, our results indicated that EZH2 interacts with TFIIIC102 and the interaction is mediated by the N-terminal region of EZH2 and the C-terminus of TFIIIC102.

### EZH2 interacts with other components of TFIIIC

**Figure 2.**
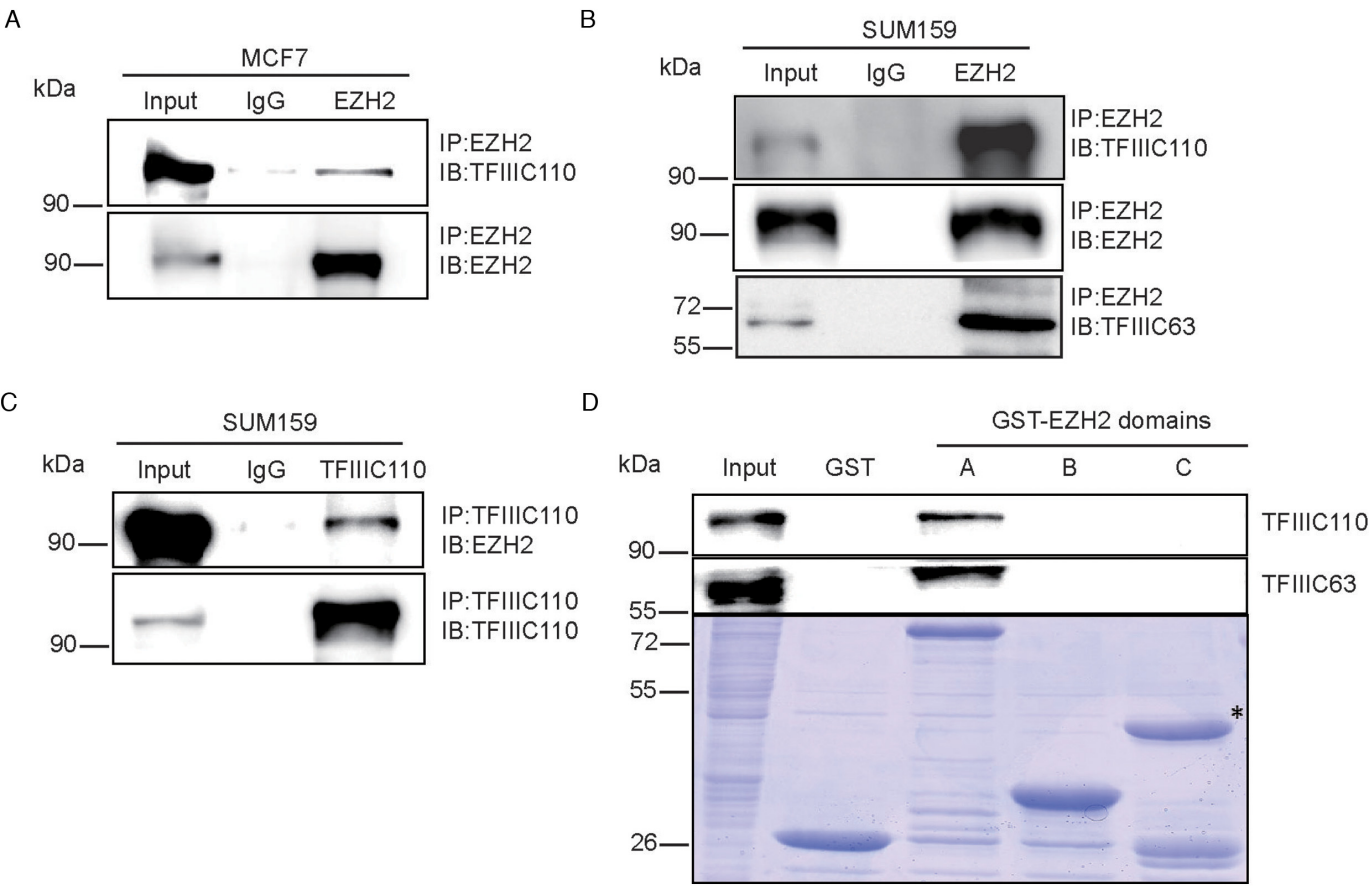
EZH2 interacts with other components of TFIIIC. (**A** and **B**) Co-IP assays were performed using MCF7 and SUM159 cell lysates with normal IgG and an EZH2 antibody. Co-immunoprecitipated proteins were detected by western blot with indicated antibodies. (**C**) Reverse Co-IP assays and western blot were performed in SUM159 cells with indicated antibodies. (**D**) GST pull-down assays were performed using three purified GST–EZH2 fragments and the HeLa cell lysates. Western blot was then conducted with indicated antibodies. The asterisk indicates fragment C of GST–EZH2.

TFIIIC complex is composed of five main subunits including TFIIIC220, TFIIIC90, TFIIIC102, TFIIIC110 and TFIIIC63, which are named according to their molecular weight (33). To examine whether EZH2 may also interact with other components of TFIIIC complex we performed protein interaction assays. In an endogenous co-immunoprecipitation analysis in MCF7 cells, we demonstrated that EZH2 was able to interact with TFIIIC110 (Figure 2a). Likewise, TFIIIC63 and TFIIIC110 were co-immunoprecipitated by an EZH2 antibody in SUM159 cells, suggesting that interaction between EZH2 and components of TFIIIC was not limited in a single cell line (Figure 2b). Furthermore, a reverse Co-IP was performed in SUM159 cells and again we found that EZH2 was co-immunoprecipited by a TFIIIC110 antibody (Figure 2c). We then performed GST pull-down assays using EZH2 GST fusion proteins. The results showed that TFIIIC63 and TFIIIC110 interacted with the N-terminal domains (Domain I and Domain II, aa 1–522) of EZH2, but not the Cys or SET domain (Figure 2d). Taken together, our results clearly indicated that EZH2 interacts with TFIIIC complex and the interaction between them is mainly mediated by the N-terminal domains of EZH2.

### EZH2 and H3K27me3 are found at the promoters of TFIIIC target genes

**Figure 3.**
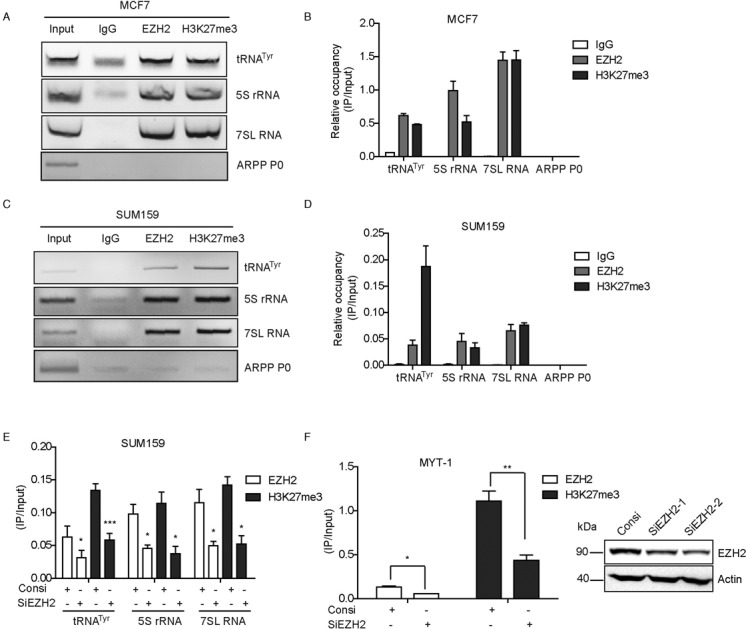
EZH2 and H3K27me3 are found at the promoters of TFIIIC target genes. (**A** and **C**) Displayed were the products of qPCR from the ChIP assays analyzed in agrose gel. ChIP assays were performed using EZH2 and H3K7me3 antibodies with indicated primers. Normal mouse IgG was used as negative control. (**B** and **D**) ChIP assays using MCF7 or SUM159 lysates were carried out followed by detection by qPCR. Results were presented by mean ± S.D. for three independent experiments. (**E** and **F**) SUM159 cells were transfected with the specific EZH2 siRNA 2 or control siRNA. Results of ChIP assays were detected by qPCR for TFIIIC targets including tRNA^Tyr^, 5S rRNA, 7SL RNA and MYT-1. MYT-1 is a known EZH2 target and was used as a positive control. Knockdown efficiency was detected by western blot with an EZH2 antibody. Results were presented by mean ± S.D. for three independent experiments. *P < 0.05, **P < 0.01 and ***P < 0.001 by Student's *t*-test.

The aforementioned findings identified interaction between EZH2 and TFIIIC complex. We therefore asked what role EZH2 may play when it interacts with TFIIIC. First, we found that knockdown of EZH2 by small interfering RNA (siRNA) did not influence the expression of TFIIIC components in neither of the two breast cancer cell lines MCF7 and SUM159 (Supplementary Figure S2a and b), indicating EZH2 could not regulate TFIIIC expression. EZH2 has been reported to mediate trimethylation of histone 3 lysine 27 at the target gene promoters, thereby leading to chromatin condensation and transcriptional repression (6,7). This raised a question whether EZH2 can be recruited to the promoters of target genes transcribed by Pol III RNA polymerase. To answer this important question, we conducted chromatin immunoprecipitation (ChIP) assays with specific EZH2 and H3K27me3 antibodies in MCF7 and SUM159 cells. EZH2 and H3K27me3 were found obviously enriched at the promoter of Pol III transcribed genes including tRNA^Tyr^, 5S rRNA and 7SL RNA (Figure 3a and c). In contrast, EZH2 and H3K27me3 was not found at the small RNA gene ARPP P0 which serves as a negative control, indicating that the ChIP assay was specific (Figure 3a and c). ChIP assays with MCF7 or SUM159 lysates were examined by both qPCR and agarose gel electrophoresis (Figure 3b and d). To validate the specificity of the ChIP assays, we knocked down EZH2 by specific siRNA in the two breast cancer cell lines mentioned above and examined the occupancy of EZH2 and H3K27me3 again. Apparently, EZH2 and H3K27me3 occupancy were greatly decreased at the promoter of MYT-1 gene, a well-established EZH2 target which served as a positive control (34,35), as well as at promoters of Pol III transcribed genes in EZH2 knockdown cells (Figure 3e and f). The above results suggested that EZH2 and H3K27me3 marks are present at Pol III transcribed genes.

### Knockdown of TFIIIC components leads to reduced EZH2 occupancy on TFIIIC targets

**Figure 4.**
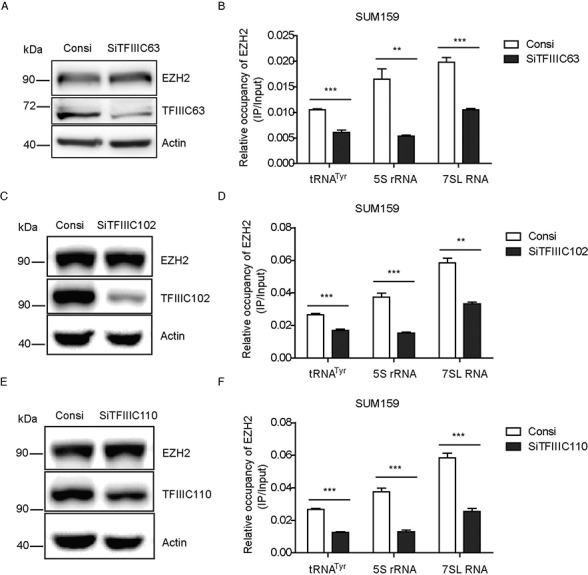
Knockdown of TFIIIC components leads to reduced EZH2 occupancy on TFIIIC targets. (**A**, **C** and **E**) SUM159 cells were transfected with specific siRNAs for TFIIIC63, TFIIIC102 or TFIIIC110. Western blots with indicated antibodies showed the knockdown efficiency and effects of siRNAs on EZH2 expression. (**B**, **D** and **F**) ChIP assays were performed with an EZH2 antibody in these TFIIIC knockdown cells, followed with qPCR using indicated primers. Results were presented by mean ± S.D. from representative of three independent experiments. **P < 0.01, ***P < 0.001 by Student's *t*-test.

To examine whether occupancy of EZH2 at Pol III targets can be affected by TFIIIC complex, components of TFIIIC complex including TFIIIC63, TFIIIC102 and TFIIIC110 were knocked down separately with small interfering RNAs and then ChIP assays using an EZH2 antibody were performed followed by qPCR analysis. Results showed that EZH2 occupancy on Pol III transcribed genes was largely reduced upon TFIIIC knockdown (Figure 4b, d and f). However, the expression of EZH2 protein was not affected by TFIIIC knockdown via siRNAs (Figure 4a, c and e). These data suggested that the reduced occupancy of EZH2 was caused by reduced recruitment by TFIIIC but not attenuated expression of the EZH2 protein. As a support, we also analyzed ChIP-seq data of EZH2, H3K27me3 and TFIIIC from the UCSC database to examine whether TFIIIC targets were co-occupied by EZH2. Not surprisingly, 42.5% TFIIIC sites were co-occupied by EZH2, with 13.3% of them co-occupied by both EZH2 and H3K27me3 (Supplementary Figure S4a). Taken together, our findings indicated that EZH2 occupancy at Pol III transcribed genes can be affected by the expression of TFIIIC complex.

### EZH2 exhibits a higher occupancy at promoters of inactive tRNA genes than that of active tRNA genes

**Figure 5.**
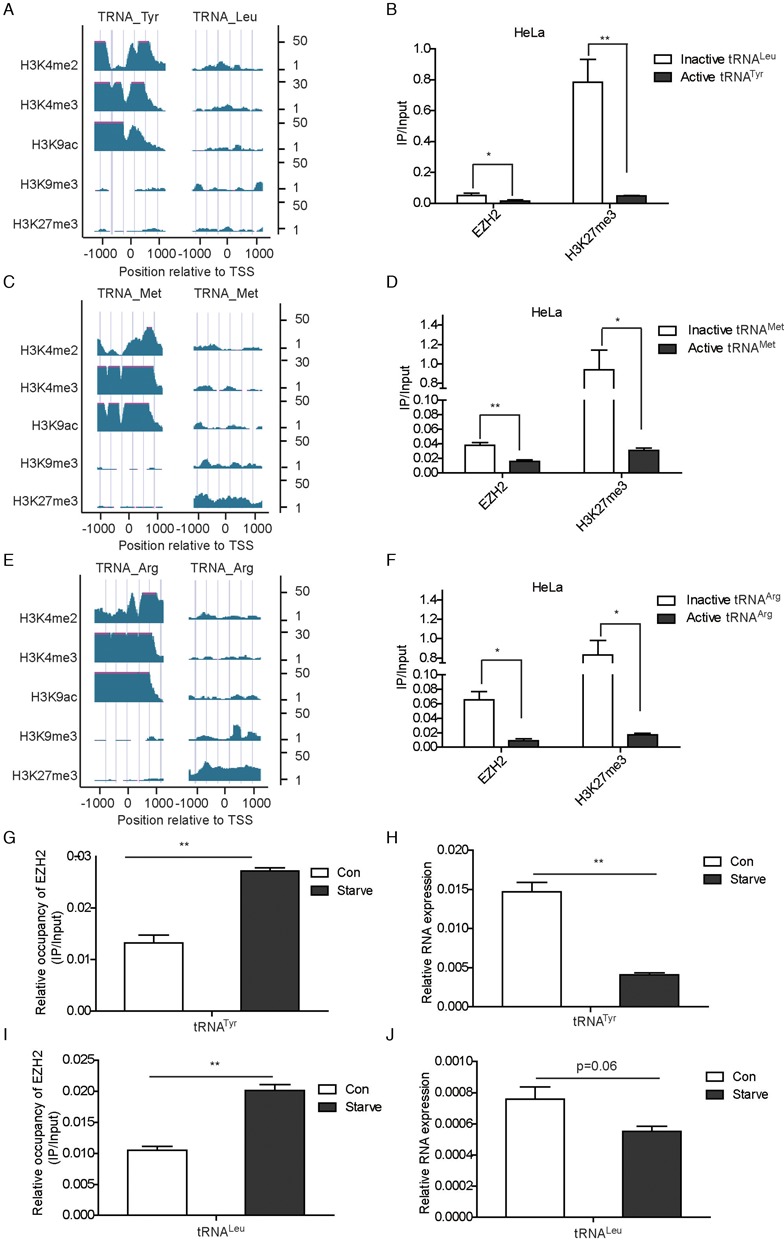
EZH2 exhibits a higher occupancy at promoters of inactive tRNA genes than that of active tRNA genes. (**A**, **C** and **E**) Displayed is the relative occupancy of indicated epigenetic marks at the promoter of the active *TRNA_Tyr* gene, the inactive *TRNA_Leu* gene, or the inactive and active *TRNA_Met* and *TRNA_Arg* genes in HeLa cells. The ChIP-seq data of the epigenetic marks in HeLa cells were acquired and analyzed from UCSC database. The scales on the right side of the graphs indicated relative peak heights for each epigenetic mark. (**B**, **D** and **F**) ChIP assays were performed in HeLa cells using EZH2 and H3K27me3 antibodies, followed by qPCR assays with indicated primers. Results were presented by mean ± S.D. from three independent experiments. *P < 0.05, **P < 0.01 by Student's *t*-test. (**G** and **I**) HeLa cells were starved for 16 h. ChIP assays were performed using control (10% FBS) and starved (0% FBS) HeLa cells with indicated antibodies, followed by qPCR to examine relative occupancies of EZH2 and H3K27me3 on indicated genes. Results were presented by mean ± S.D. from representative of three independent experiments. **P < 0.01 by Student's *t*-test. (**H** and **J**) Expression of tRNA^Tyr^ and tRNA^Leu^ were examined by qPCR. Results were presented by mean ± S.D. from representative of three independent experiments. **P < 0.01 by Student's *t*-test.

Different transcripts of tRNA are known to be transcribed at different efficiency. Some of the transcripts are actively transcribed and their promoters are usually occupied by Pol III, transcriptional factors and related epigenetic marks including H3K4me2, H3K4me3 or H3K9ac. These tRNA genes harboring transcription-promoting marks H3K4me2, H3K4me3 or H3K9ac are called active tRNAs. Whereas some of the tRNA transcripts are slowly transcribed and their promoters are occupied by repressive epigenetic marks, e.g. H3K9me3 and H3K27me3 (26). These tRNAs with H3K9me3 and H3K27me3 marks at their promoters are called inactive tRNAs. To uncover the relationship between EZH2 and tRNA transcription, ChIP-seq datasets from ENCODE database at UCSC were retrieved and analyzed. Relative occupancy for a panel of epigenetic marks at promoters of the two different types of tRNA transcripts (inactive and active tRNAs) were displayed and compared with that of EZH2. In sharp contrast to the active epigenetic marks including H3K4me2, H3K4me3 and H3K9ac marks and similar to the inactive epigenetic marks at the promoter of the inactive tRNA gene *TRNA_Tyr* and the active tRNA gene *TRNA_Leu* (Figure 5a), the occupancy of EZH2 and H3K27me3 was significantly higher at the promoter of the inactive tRNA than that of the active tRNA in HeLa cells (Figure 5b). A Similar tendency of EZH2 and H3K27me3 occupancy at promoters of inactive or active tRNAs was also observed in MCF7 and SUM159 cells (Supplementary Figure S5a and b). These results suggested that EZH2 might be recruited to promoters of Pol III transcribed genes to suppress gene transcription. To validate this hypothesis, we examined the occupancy of EZH2 and H3K27me3 at promoters of another two pairs of active and inactive tRNA genes. Likewise, the relative occupancy of EZH2 and H3K27me3 at promoters of inactive or active *TRNA_Met* and *TRNA_Arg* genes were almost identical to that of *TRNA_Tyr* and *TRNA_Leu* genes (Figure 5c–f).

To further investigate the correlation of EZH2 occupancy and tRNA expression, HeLa cells were serum-starved for 16 h and ChIP assays were performed using an EZH2 antibody. The occupancy of EZH2 on different tRNAs was detected by qPCR. Also, we harvested protein and RNA of control (10% FBS) and serum-starved (0% FBS) HeLa cells and checked the expression of EZH2 and tRNAs. We found that when HeLa cells were serum-starved, the expression of tRNAs and EZH2 were both decreased (Supplementary Figure S5c, Figure 5h and j). However, the occupancy of EZH2 on different tRNAs was increased upon serum starvation (Figure 5g and i). There is a possible explanation that when cells are short of nutrition, they decrease the rate of protein synthesis by increasing recruitment of negative regulators (e.g. EZH2) to tRNA genes. Collectively, these results indicated for the first time that EZH2 interacts with TFIIIC and is involved in the transcriptional repression of Pol III target genes

### EZH2 suppresses transcription of Pol III target genes

**Figure 6.**
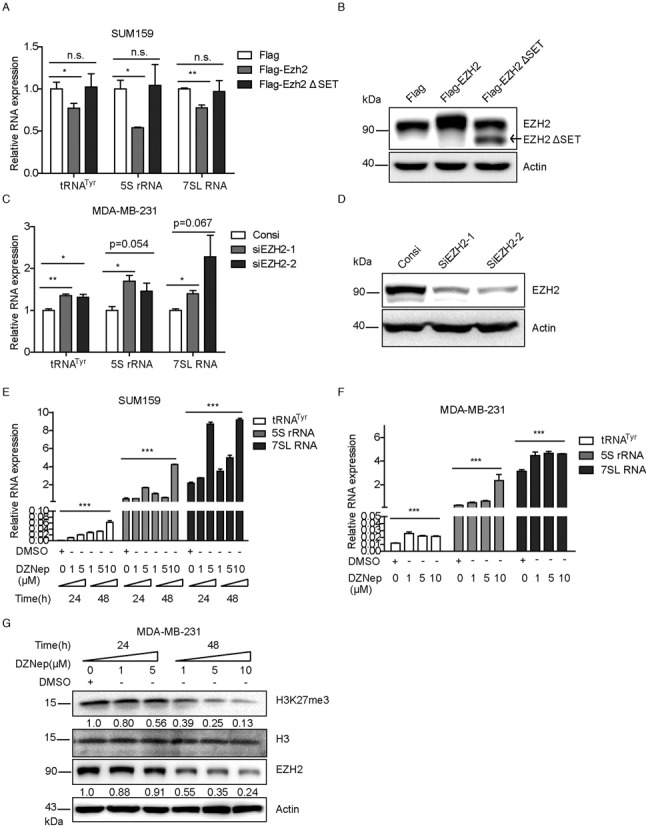
EZH2 suppresses the transcription of Pol III target genes. (**A**) SUM159 cells were transfected with Flag-EZH2 or Flag-EZH2ΔSET mutant or the control vectors for 48 h. Quantitative PCR showed the relative expression of indicated RNA genes. (**B**) The expression level of EZH2 was detected using western blot probed by indicated antibody. The arrow indicates transfected Flag-EZH2ΔSET mutant. (**C**) MDA-MB-231 cells were transfected with two different EZH2 siRNAs or control siRNA. Quantitative PCR was performed to examine the expression of tRNA^Tyr^, 5S rRNA and 7SL RNA. ARPP P0 was used as the reference control. Results were presented by mean ± S.D. from three independent experiments. *P < 0.05, **P < 0.01 by Student's *t*-test. (**D**) The efficiency of EZH2 knockdown by siRNA was determined by western blot probed by an EZH2 antibody. (**E**) SUM159 cells were treated with DZNep or DMSO for 24 or 48 h with indicated concentrations. The expression of tRNA^Tyr^, 5S rRNA and 7SL RNA was examined by qPCR. Displayed were representative autographs of three independent experiments and were presented by mean ± S.D. ***P < 0.001 by one-way ANOVA. (**F**) MDA-MB-231 cells were treated with DZNep or DMSO for 48 h and the expression of tRNA^Tyr^, 5S rRNA and 7SL RNA was examined by qPCR. Displayed were representative autographs of three independent experiments and were presented by mean ± S.D. ***P < 0.001 by one-way ANOVA. (**G**) Effects of DZNep on EZH2 and H3K27me3 expression were detected by western blot with indicated antibodies. Actin and H3 were used as reference controls for EZH2 and H3K27me3 respectively.

The presence of EZH2 and H3K27me3 at promoters of Pol III transcribed genes, especially on the inactive genes, strongly suggested that EZH2 may regulate Pol III directed gene transcription through trimethylation of histone H3 at lysine 27. To test this hypothesis we examined the role of EZH2 in both gain- and loss-of -function experiments. For gain-of-function experiment, ectopic expression of wild-type EZH2 led to reduced production of Pol III target genes while the Flag-EZH2ΔSET mutant displayed no significant effect on Pol III transcription (Figure 6a and b). To rule out the possibility that deletion of the whole SET domain could impair function of EZH2 other than its catalytic activity, we also constructed the catalytic inactive mutant of EZH2, the EZH2 F667I plasmid. Forced expression of this mutant of EZH2 showed no affection on Pol III target expression (Supplementary Figure S6a and b), suggesting that EZH2 methyltransferase activity is required for suppression of Pol III target gene transcription. Furthermore, knockdown of endogenous EZH2 in MDA-MB-231 cells by two different siRNAs led to increased expression of Pol III targets, tRNA^Tyr^, 5S rRNA and 7SL RNA (Figure 6c and d). These data suggested that EZH2 plays a suppressive role on the Pol III target gene transcription.

The aforementioned findings suggested a requirement of EZH2 methyltranferase activity for inhibition of Pol III target gene transcription. We therefore scrutinized this possibility by using DZNep, a commonly used potent EZH2 inhibitor (36). DZNep has been reported to mainly inhibit the methyltranferase activity of EZH2. However, when DZNep reaches a certain concentration, it may also affect EZH2 expression. We treated SUM159 and MDA-MB-231 cells with DZNep or DMSO for various time and concentrations. Western blot analysis showed that the expression of EZH2 was slightly downregulated by DZNep treatment in 24 h but was robustly reduced in 48 h (Figure 6g). Trimethylation of H3K27 was reduced in a dose- and time-dependent manner when cells were treated with DZNep (Figure 6g). In addition, the expression of all these three Pol III transcribed genes was upregulated in a dose dependent manner when the concentration of DZNep is below 5 μM. However, the expression of these three Pol III transcribed genes did not show a further increase when the concentration of DZNep reached a high dose of 10 μM (Figure 6e and f). This might be due to the high concentration of DZNep affects gene expression other than EZH2 activity. Taken together, these results indicated that EZH2 is involved in Pol III target gene repression through trimethylation of H3K27 at their promoters.

### PRC2 component SUZ12 also interacts with TFIIIC complex and affects the function of EZH2 on Pol III transcription by maintaining EZH2 stability

**Figure 7.**
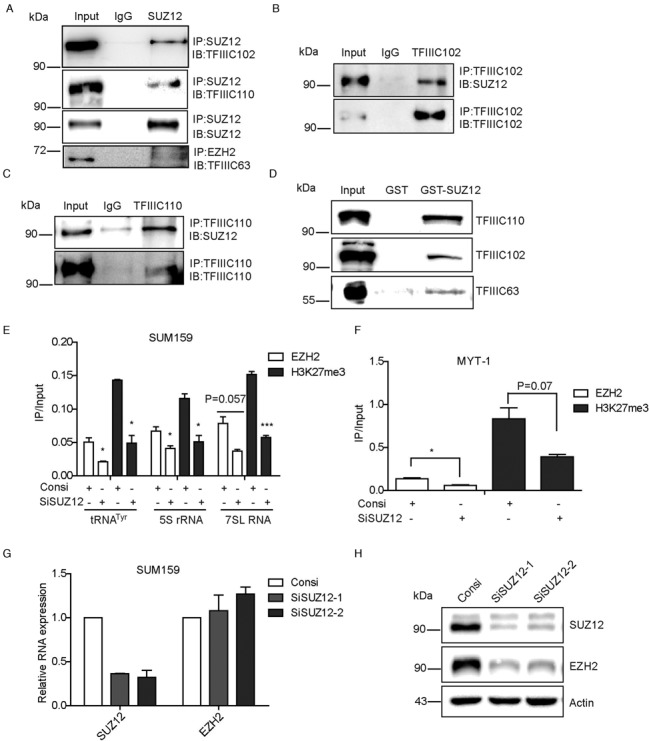
PRC2 component SUZ12 also interacts with TFIIIC complex and affects the function of EZH2 on Pol III transcription by maintaining EZH2 stability. (**A**) Co-IP assays were performed using SUM159 cell lysate with IgG and SUZ12 antibodies and detected by western blot with indicated antibodies. (**B** and **C**) Reverse Co-IP assays were performed with TFIIIC102 and TFIIIC110 antibodies and detected by western blot with indicated antibodies. (**D**) GST pull-down assays were carried out with GST-SUZ12 protein and HeLa cell lysates, detected by western blot with indicated antibodies. (**E**–**H**) SUM159 cells were transfected with SUZ12 siRNA 2 or control siRNA for 48 h; ChIP assays were performed with indicated antibodies to detect EZH2 and H3K27me3 occupancy. (e) Occupancy of EZH2 and H3K27me3 at promoters of tRNA^Tyr^, 5S rRNA and 7SL RNA genes were detected by qPCR with indicated primers. (**F**) As a positive control, the EZH2 and H3K27me3 occupancy was examined at MYT-1 promoter by qPCR after ChIP assay. (g and h) Quantitative PCR and western blot assays were conducted to examine the knockdown efficiency of SUZ12 and its effect on EZH2 expression. Results were presented by mean ± S.D. from three independent experiments. *P < 0.05, *** *P* < 0.001 by Student's *t*-test.

SUZ12 has been indicated to play a role in guiding the PRC2 complex to the target gene promoters and is essential for the methylation function of PRC2 complex (37,38). Given that EZH2 affects Pol III mediated gene transcription in a methyltransferse activity dependent manner, we wanted to know whether PRC2 component SUZ12 is involved in this function. To this end, co-immunoprecipitation was performed with a specific SUZ12 antibody using lysates from SUM159 cells. Results indicated that TFIIIC components including TFIIIC63, TFIIIC102 and TFIIIC110 were able to associate with endogenous SUZ12 (Figure 7a). Reversely, both TFIIIC102 and TFIIIC110 were able to associate with SUZ12 endogenously in SUM159 cells (Figure 7, b and c). SUZ12 was also found to associate with TFIIIC63, TFIIIC102 and TFIIIC110 in a GST pull-down assay using purified GST-SUZ12 (Figure 7d). Furthermore, we performed ChIP assays with an SUZ12 antibody. Like EZH2, SUZ12 was found at all the Pol III targets examined (Supplementary Figure S7a). These data clearly demonstrated that SUZ12 also interacts with TFIIIC complex and was present at Pol III targets together with EZH2.

To test whether SUZ12 is required for trimethylation of H3K27 by EZH2 at promoters of Pol III transcribed genes, we transfected SUM159 cells with control or SUZ12 siRNAs and then performed ChIP assays to detect the occupancy of EZH2 and H3K27me3 at the target gene promoters. Interestingly, knockdown of SUZ12 greatly affected EZH2 and H3K27me3 occupancy at the promoter of Pol III target genes (Figure 7e), suggesting that SUZ12 is required for EZH2 and H3K27me3 occupancy at the target gene promoters. Similarly, in a control experiment depletion of SUZ12 led to reduced occupancy of EZH2 and H3K27me3 at the promoter of target gene MYT-1 (Figure 7f). The efficiency of SUZ12 knockdown was examined by both qPCR (Figure 7g) and western blot analysis (Figure 7h). Intriguingly, knockdown of SUZ12 also led to a robust reduction of EZH2 protein level without affecting its transcription (Figure 7g and h), suggesting that SUZ12 is required for the stability of PRC2 complex. Likewise, knockdown of EZH2 also led to a reduced SUZ12 protein expression (Supplementary Figure S3a and b). Interestingly, overexpression of SUZ12 could affect neither the expression of EZH2 nor Pol III targets, suggesting SUZ12 does not directly regulate the expression of Pol III targets (Supplementary Figure S7b and c). Collectively, PRC2 component SUZ12 also interacts with TFIIIC complex and is required for EZH2-mediated transcriptional suppression of Pol III transcribed genes by maintaining the stability of EZH2.

## DISCUSSION

**Figure 8.**
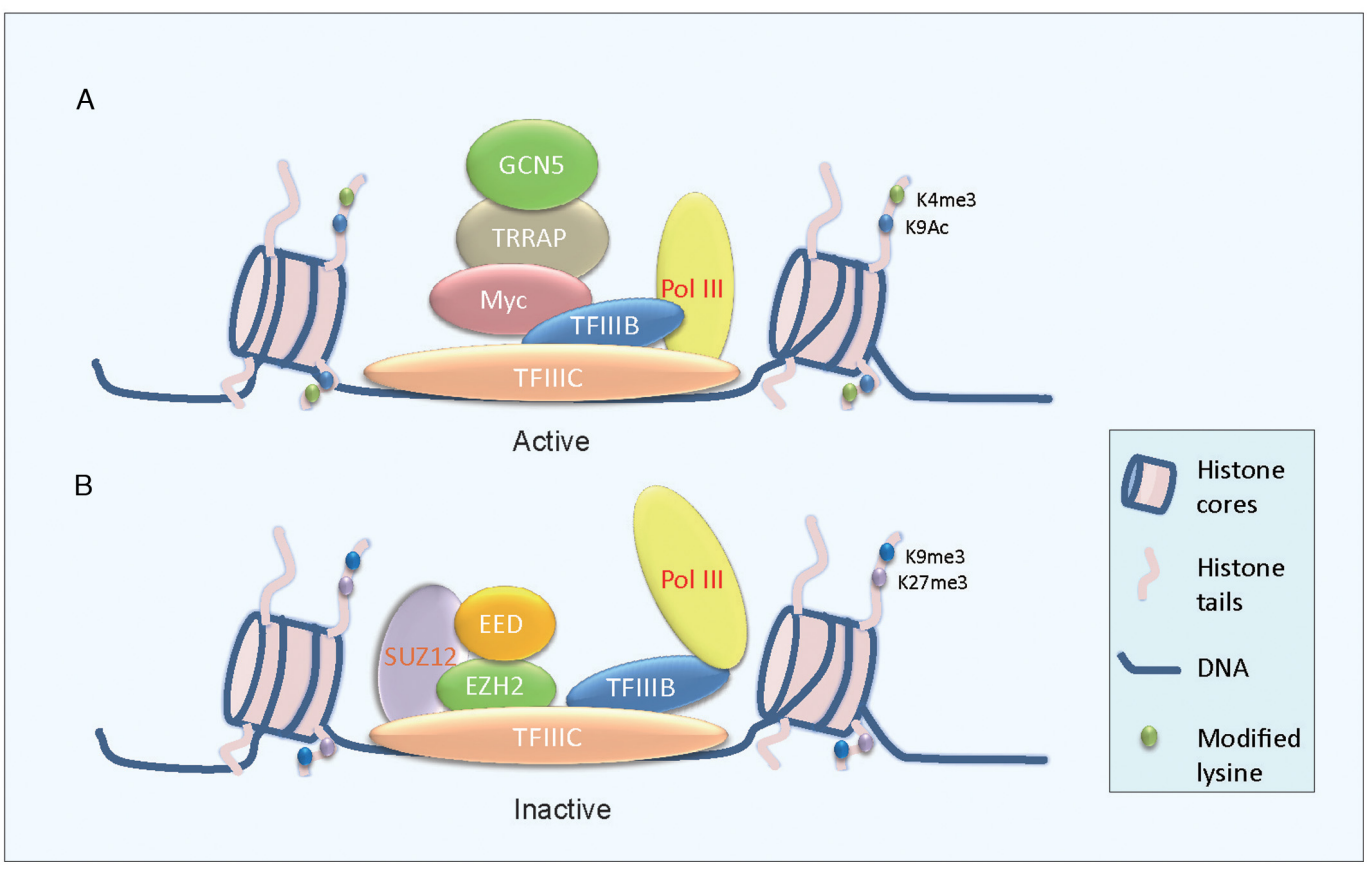
A working model for PRC2 regulation of Pol III transcription. (**A**) A model depicting the role of c-Myc regulation of Pol III transcription through recruiting acetyltransferase TRRAP and GCN5, consisting one of the mechanisms accounting for activation of Pol III transcription (17,18). (**B**) A proposed model based on findings in the present investigation that PRC2 component EZH2 interacts with TFIIIC complex and is recruited to the promoter of target non-translated RNA genes. EZH2 then trimethylates H3K27 and forms a suppressive complex and represses target non-translated RNA transcription.

In the present report we uncovered a novel role of EZH2, as a key epigenetic regulator, in controlling transcription of a subset of small RNA genes transcribed by RNA polymerase III including tRNA, 5S rRNA and 7SL RNA. EZH2 has been known to repress the expression of RNA polymerase II transcribed genes (39–43). For the first time we identified that EZH2 is linked with RNA polymerase III transcription by demonstrating its interaction with members of a transcriptional factor complex, TFIIIC. In a mass spectrometry assay, we first pinpointed that EZH2 via its N-terminal domains interacts with TFIIIC102, a subunit of TFIIIC complex. Given that TFIIIC functions as an integrated complex, we then examined whether EZH2 also interacts with other components of TFIIIC complex. We found that EZH2 interacts with TFIIIC110 and TFIIIC63. Moreover, EZH2 and its substrate H3K27me3 were found present at the promoters of non-translated RNAs including tRNA^Tyr^, 5S rRNA and 7SL RNA. It was known that epigenetic marks H3K4me2, H3K4me3 and H3K9ac were enriched at the promoters of active tRNA genes; whereas at the promoters of inactive tRNA genes, epigenetic marks H3K27me3 and H3K9me3 were enriched (26). In light of these findings we built up a model depicting that EZH2 was involved in Pol III transcription through interaction with TFIIIC and there EZH2 transforms its surrounding histone H3K27 to the trimethylated form and suppresses target gene expression (Figure 8). This model was supported by the results that knockdown of TFIIIC components led to a reduced occupancy of EZH2 at these promoters. We also found that SUZ12 could interact with different components of TFIIIC complex and knockdown of SUZ12 decreased the occupancy of EZH2 and H3K27me3 at promoters of Pol III transcribed genes. Furthermore, the reduction in the protein level of EZH2 upon SUZ12 knockdown was not caused by regulation of EZH2 at the transcriptional level (Figure 7g), suggesting that SUZ12 is required for maintaining the stability of EZH2 within PRC2 complex. In order to examine whether SUZ12 was able to regulate the transcription of Pol III targets directly, SUZ12 was forced expressed in SUM159 cells. Surprisingly, although knockdown of SUZ12 led to an obvious decrease in EZH2 protein level, neither the expression of EZH2 nor Pol III targets was affected upon overexpression of SUZ12. These results indicated that SUZ12 is not directly involved in the regulation of Pol III transcription. Therefore, the functional consequences of the interaction between SUZ12 and EZH2 may be context-dependent. Interestingly, a decrease of SUZ12 expression in protein level was also observed in EZH2 knockdown cells. We thus proposed that both EZH2 and SUZ12 are required for maintaining the stability of PRC2 complex during the regulation of Pol III transcribed non-translated RNA. However, we did not find interaction between the PRC2 component EED with TFIIIC complex (data not shown).

Epigenetic regulation of RNA polymerase II has been well established. However, epigenetic regulation of transcription mediated by RNA polymerase III is still unclear although researchers have found epigenetic marks are also present at promoters of Pol III transcribed genes. C-Myc, for example, was found to be able to trigger Pol III transcription through recruiting acetyltransferase GCN5 and TRRAP (17) (Figure 8a). H3Ser10 phosphorylation was also found to be associated with increased TFIIIB expression and Pol III target transcription (44). These findings indicated that epigenetic regulation is involved in Pol III transcription. However, it remains unclear how epigenetic events regulate Pol III transcribed target gene expression. In the present study we presented a novel paradigm by demonstrating that EZH2 suppresses Pol III transcribed target gene expression by catalyzing H3K27 trimethylation at the gene promoters through interaction with TFIIIC complex (Figure 8b). However, as epigenetic regulation is complicated, we do not exclude possibility that other epigenetic marks and regulators may co-occupancy Pol III transcribed small RNA genes with EZH2. Given that histone modifications often regulate gene transcription cooperatively, we anticipate that another repressive epigenetic mark H3K9me3, like H3K27me3, may also be involved in the repression of non-translated RNA genes transcribed by Pol III. Methyltransferase G9a and GLP are found to cooperate with PRC2 to maintain gene silencing, therefore it is highly possible that G9a and GLP also regulate Pol III transcribed genes (45). To this end, possible interaction of G9a or GLP with TFIIIC complex warrants future investigations.

It was demonstrated that forced reduction of c-Myc expression results in increased expression of H3K27me3 mark in mouse and human prostate cancers (46). Meanwhile, pRB family proteins were found to be required for H3K27 trimethylation and polycomb binding to and silencing tumor suppressor *p16INK4a* (47). Further, c-Myc was known to activate Pol III transcription (18,21) and pRB was reported to represses Pol III transcription (18,21). These findings suggested that occupancy of EZH2 and H3K27me3 on Pol III transcribed genes may be regulated by oncogenic or tumor suppressive proteins including c-Myc and pRB directly or indirectly.

It was reported that the expression level of TFIIIC components and production of tRNAs were both elevated in tumors than in normal tissues, noted that tumors require more materials for protein synthesis and cell growth (48,49). Generally speaking the epigenetic regulation of tRNA synthesis in normal or pathological conditions still remains unclear. Therefore, it would be interesting to find the role of EZH2-mediated epigenetic regulation in transcription of Pol III transcribed genes in physiological or pathological conditions, especially in cancers.

In summary, we demonstrated that PRC2 component EZH2 and SUZ12 repress RNA polymerase III transcribed non-translated RNA gene transcription through interacting with and being recruited by transcriptional factor IIIC. Both EZH2 and H3K27me3 are present at the promoters of Pol III target genes and suppress their expression. Our findings provide evidence that PRC2 complex regulate RNA polymerase III transcribed gene expression in mammalian cells.

## SUPPLEMENTARY DATA

Supplementary Data are available at NAR Online.

SUPPLEMENTARY DATA
