# Supplementary material for: PRC2 regulates RNA polymerase III transcribed non-translated RNA gene transcription through EZH2 and SUZ12 interaction with TFIIIC complex
Source: Nucleic Acids Res. 2015 Jun 1;43(13):6270–84. doi: 10.1093/nar/gkv574 (PMC4513857; doi:10.1093/nar/gkv574)
Supplement: SUPPLEMENTARY DATA [file supp_43_13_6270__index.html]

PRC2 regulates RNA polymerase III transcribed non-translated RNA gene transcription through EZH2 and SUZ12 interaction with TFIIIC complex — PRC2 regulates RNA polymerase III transcribed non-translated RNA gene transcription through EZH2 and SUZ12 interaction with TFIIIC complex — SUPPLEMENTARY DATA 

# PRC2 regulates RNA polymerase III transcribed non-translated RNA gene transcription through EZH2 and SUZ12 interaction with TFIIIC complex

## SUPPLEMENTARY DATA

- SUPPLEMENTARY DATA
