# Supplementary material for: PRC2 regulates RNA polymerase III transcribed non-translated RNA gene transcription through EZH2 and SUZ12 interaction with TFIIIC complex
Source: Nucleic Acids Res. 2015 Jun 1;43(13):6270–84. doi: 10.1093/nar/gkv574 (PMC4513857; doi:10.1093/nar/gkv574)
Supplement: SUPPLEMENTARY DATA [file supp_gkv574_nar-01203-x-2015-File010.pdf]

Supplementary Tables:

Table S1. Primers used for qRT-PCR

Name	Sequence
tRNA^{Tyr}	
tRNA^{Tyr} F	5-CCTTCGATAGCTCAGCTGGTAGAGCGGAGG-3
tRNA^{Tyr} R	5-CGGAATTGAACCAGCGACCTAAGGATGTCC-3
5S rRNA	
5S rRNA F	5-GGCCATACCACCCTGAACGC-3
5S rRNA R	5-CAGCACCCGGTATTCCCAGG-3
7SL RNA	
7SL RNA F	5-GTGTCCGCACTAAGTTCGGCATCAATATGG-3
7SL RNA R	5-TATTCACAGGCGCGATCCCACTACTGATC-3
SUZ12	
SUZ12 F	5-CCGAGCACTGTGGTTGAGTA-3
SUZ12 R	5-AACTGCATCTGATGGTGGTG -3

Table S2. Primers used for ChIP-qPCR

Name	Sequence
tRNA^{Tyr}	
tRNA^{Tyr} F	5-CCTTCGATAGCTCAGCTGGTAGAGCGGAGG-3
tRNA^{Tyr} R	5-CGGAATTGAACCAGCGACCTAAGGATGTCC-3
5S rRNA	
5SrRNA F	5-GGCCATACCACCCTGAACGC-3
5SrRNA R	5-CAGCACCCGGTATTCCCAGG-3
7SL RNA	
7SLRNA F	5-GTGTCCGCACTAAGTTCGGCATCAATATGG-3
7SLRNA R	5-TATTCACAGGCGCGATCCCACTACTGATC-3
Active tRNA^{Tyr}	
Active tRNA^{Tyr} F	5-GTGGCCAAGTGGTAAGGCGTC-3
Active tRNA^{Tyr} R	5-ACCCGACTTCCCCACAGCC-3
Inactive tRNA^{Leu}	
Inactive tRNA^{Leu} F	5-CTTGAAACTTGCCCCAGTCA-3
Inactive tRNA^{Leu} R	5-TTCGCGTACTTTTTAAATGCTG-3
Active tRNA^{Met}	
Active tRNA^{Met} F	5-AAGAAAGTGGTGATGCCGAGTGC-3
Active tRNA^{Met} R	5-CGTCAGTCTCATAATCTGAAGGTCGTG-3
Inactive tRNA^{Met}	
Inactive tRNA^{Met} F	5-GCGTTCCGATATTTAGTGTATCCAACT-3
Inactive tRNA^{Met} R	5-GCTCAACAGCAAGCCCTCTTAGT-3
Active tRNA^{Arg}	
Active tRNA^{Arg} F	5-CCGTCGCCTCTGGCTTAACATAG-3
Active tRNA^{Arg} R	5-GAACCTTTGAATGCCTTCAGCCTCTA-3

Inactive tRNA^{Leu}	
Inactive tRNA^{Arg} F	5-TGACCATCTCTGCCGGGACT-3
Inactive tRNA^{Arg} R	5-TGAGGTCTCTGTGGCGCAATG-3
MYT-1	
MYT-1 F	5-ACAAAGGCAGATACCCAACG-3
MYT-1 R	5-GCAGTTTCAAAAAGCCATCC-3

Supplementary Figures

Liu , et al.

Supplementary Figure 1

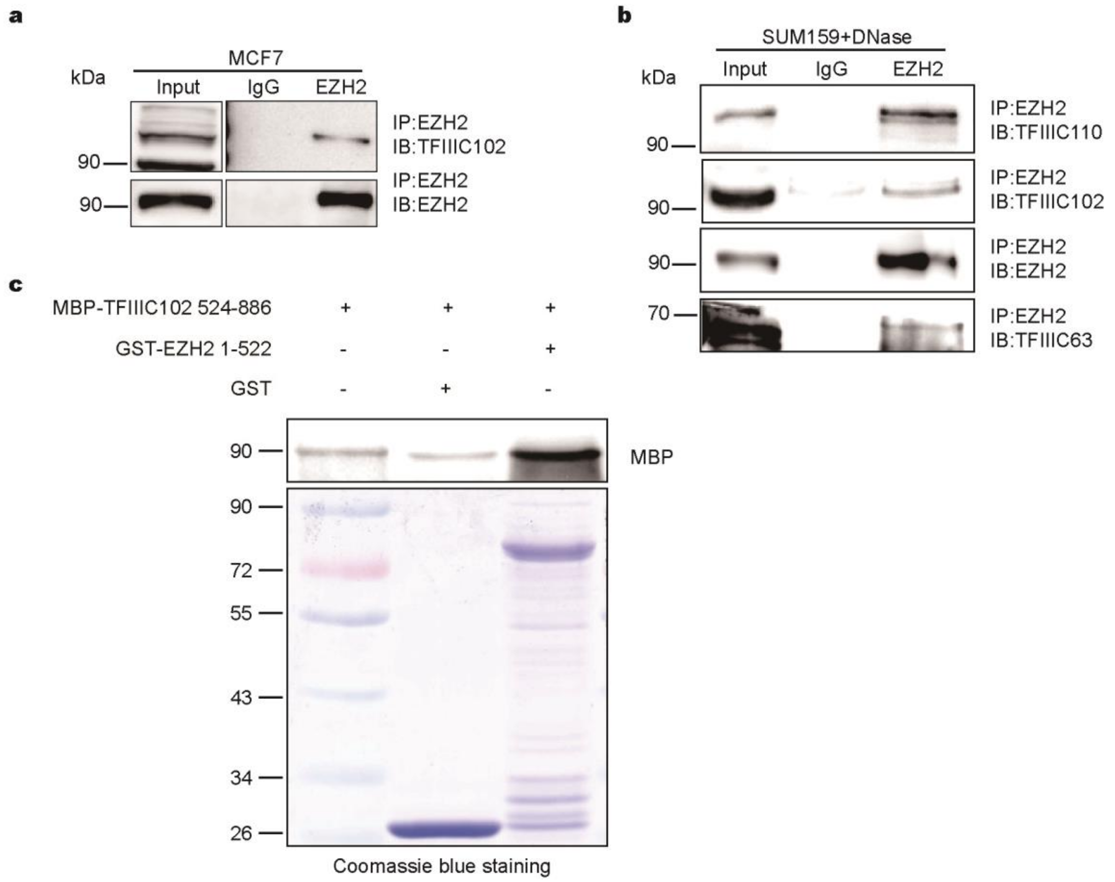

Liu , et al.

Supplementary Figure 2

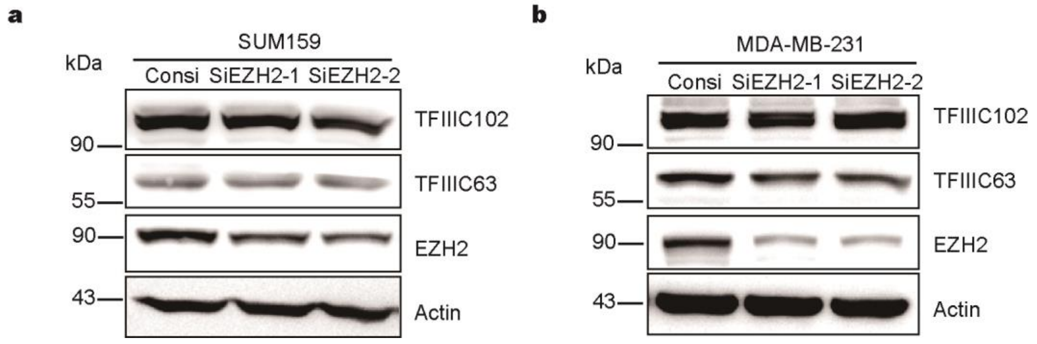

Liu , et al.

Supplementary Figure 3

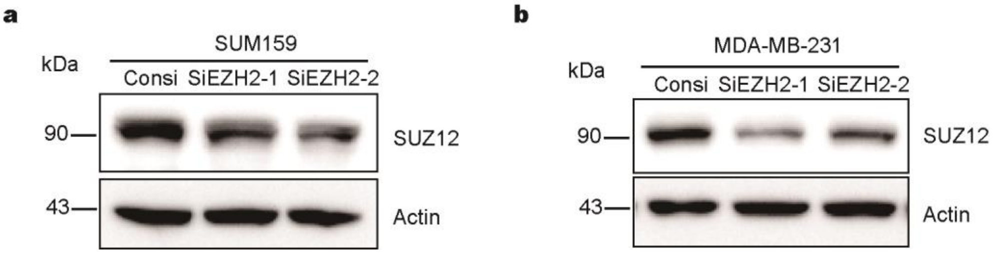

Liu , et al.

Supplementary Figure 4

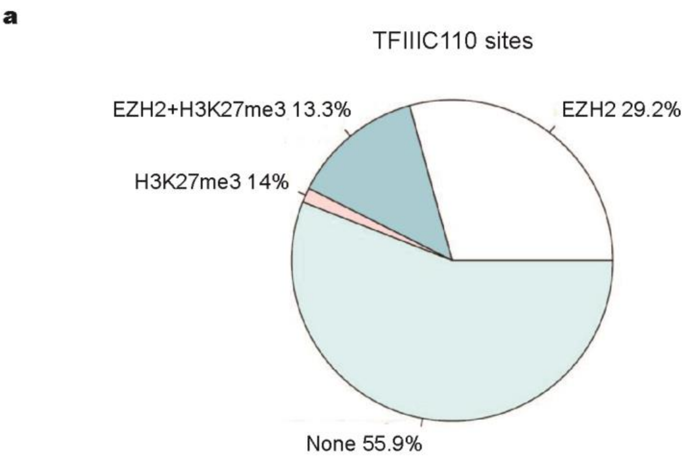

Liu , et al.

Supplementary Figure 5

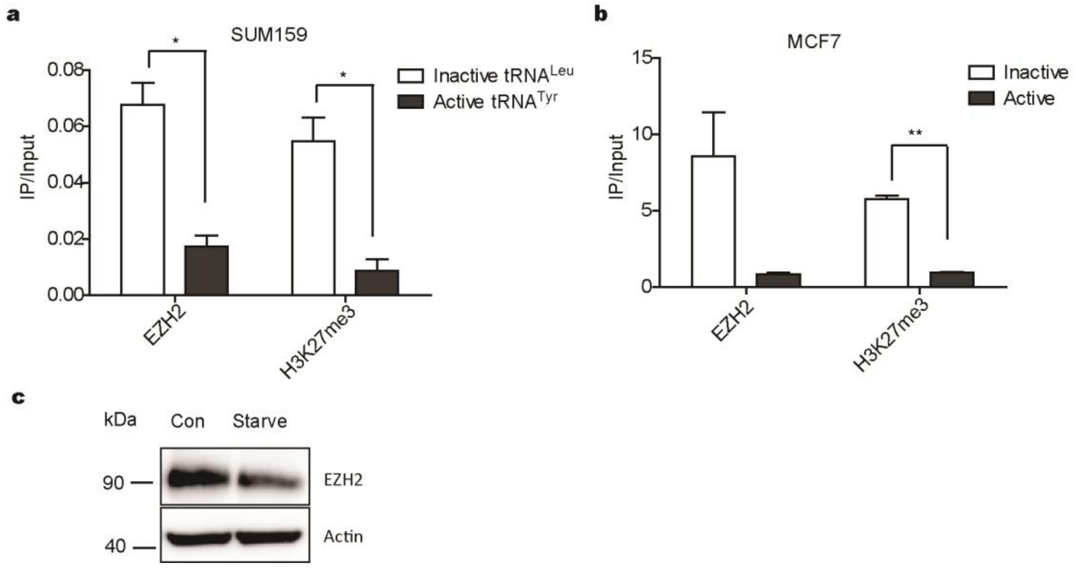

Liu , et al.
Supplementary Figure 6

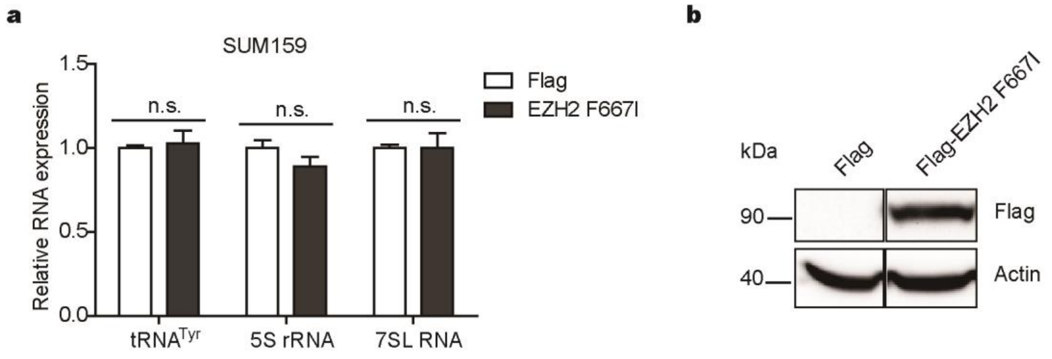

Liu , et al.
Supplementary Figure 7

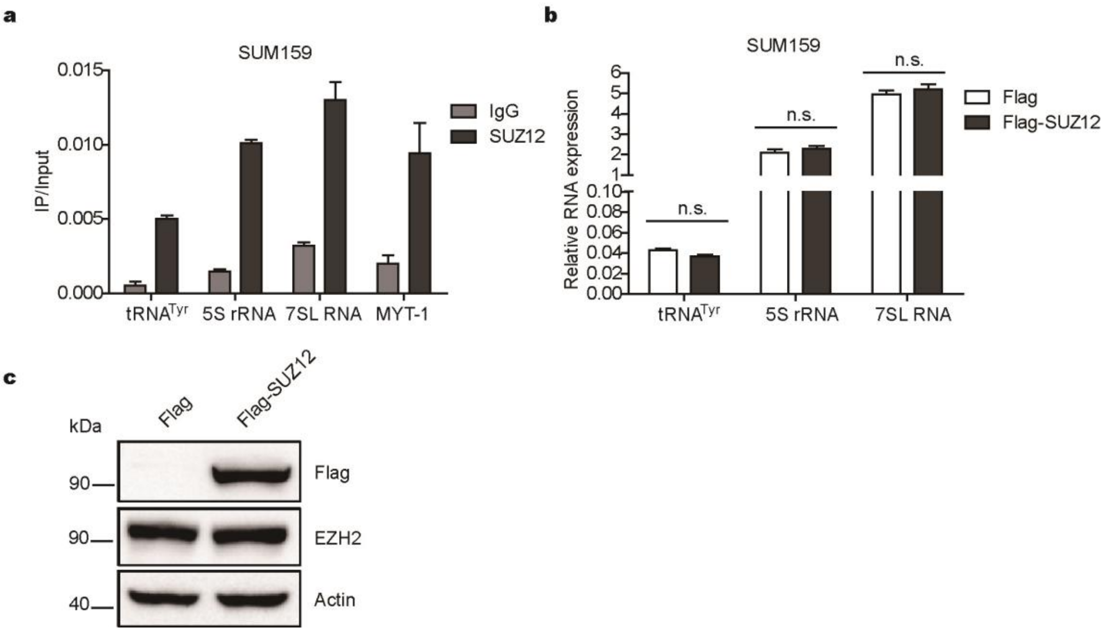

Supplementary figure legends

Supplementary Figure 1. EZH2 interacts with TFIIIC102 in MCF7 cells.

(a) Co-IP assays were performed with an alternative EZH2 antibody (Millipore) and were analyzed by Western blot with indicated antibodies in MCF7 cells. (b) Co-IP assays were performed with EZH2 antibody using lysates of SUM159 cells, which were treated with 0.15 μM DNase, and were then analyzed by Western blot with indicated antibodies. (c) GST pull-down assays were performed with purified GST, GST-EZH2(aa 1-522) and MBP-TFIIIC102 (aa 524-886) proteins. Protein interaction was detected by Western blot probed with MBP antibody. The gel was stained by Coomassie brilliant blue.

Supplementary Figure 2. EZH2 does not affect the expression of TFIIIC components.

(a, b) SUM159 and MDA-MB-231 cells were transfected with two different EZH2 siRNAs or control siRNAs. Western blot was performed to examine the levels of TFIIIC63 and TFIIIC102 with indicated antibodies.

Supplementary Figure 3. Knockdown of EZH2 led to reduced SUZ12 at protein level.

Western blot analysis showed reduced level of SUZ12 in EZH2 knockdown cells, which are SUM159 (a) and MDA-MB-231 (b).

Supplementary Figure 4. More than forty percent of TFIIIC targets were co-occupied by EZH2.

(a) Displayed is the graph showing percentage of genes co-occupied by TFIIIC and EZH2 or H3K27me3. For identifying TFIIIC binding sites co-occupied with EZH2 and H3K27me3 in HeLa cells, we used the data from GEO (GSM733696, GSM935342 and GSM1003520). Sites located within 1000 bp of each other were

considered to be co-occupied .We used bedtools v2.19.1 to find intersect regions between each binding sites. Used R for statistics and plotting analysis.

Supplementary Figure 5. EZH2 exhibits a higher occupancy on promoters of inactive tRNA genes than that of active tRNA genes.

(a, b) ChIP assays were performed in SUM159 and MCF7 cells using EZH2 and H3K27me3 antibodies, followed by qPCR assays with indicated primers. Results were presented by mean ± S.D. from three independent experiments. * $p< 0.05$, ** $p< 0.01$ by Student's *t*-test. (c) Control (10%FBS, 16 h) and serum-starved (0%FBS, 16 h) HeLa cell protein was extracted for Western blot to examine the expression level of EZH2.

Supplementary Figure 6. The catalytic inactive mutant of EZH2 Flag-EZH2 F667I does not affect the expression of Pol III targets.

(a) SUM159 cells were transfected with the catalytic inactive mutant of EZH2, Flag-EZH2 F667I and the control vector for 48 h. Quantitative PCR showed the relative expression of indicated RNA genes. (b) The level of EZH2 was detected using Western blot probed by indicated antibody.

Supplementary Figure 7. SUZ12 does not directly affect transcription of Pol III targets.

(a) ChIP assays were performed using ChIP-grade SUZ12 antibody, followed by qPCR assays with indicated primers. Results were presented by mean ± S.D. from a representative experiment. (b) SUM159 cells were transfected with Flag-SUZ12 or vector plasmids. Quantitative PCR assays were performed with indicated primers to examine the expression of Pol III targets. (c) Western blot assays were performed to detect the level of SUZ12 and EZH2 with indicated antibodies.
